# Comparative Role of rGO, AgNWs, and rGO–AgNWs Hybrid Structure in the EMI Shielding Performance of Polyaniline/PCL-Based Flexible Films

**DOI:** 10.3390/molecules30244693

**Published:** 2025-12-08

**Authors:** Brankica Gajić, Marija Radoičić, Muhammad Yasir, Warda Saeed, Silvester Bolka, Blaž Nardin, Jelena Potočnik, Gordana Ćirić-Marjanović, Zoran Šaponjić, Svetlana Jovanović

**Affiliations:** 1 “Vinča” Institute of Nuclear Sciences, National Institute of Republic of Serbia, University of Belgrade, Mike Petovića Alasa 12-14, 11000 Belgrade, Serbia; brankica.gajic@vin.bg.ac.rs (B.G.); jpotocnik@vin.bg.ac.rs (J.P.); svetlanajovanovic@vin.bg.ac.rs (S.J.); 2 Division of Microrobotics and Control Engineering, Department of Computing Science, Carl von Ossietzky Universität Oldenburg, 26129 Oldenburg, Germany; muhammad.yasir@uni-oldenburg.de (M.Y.); warda.saeed@uni-oldenburg.de (W.S.); 3 Faculty of Polymer Technology, Ozare 19, 2380 Slovenj Gradec, Slovenia; silvester.bolka@ftpo.eu (S.B.); blaz.nardin@ftpo.eu (B.N.); 4 Faculty of Physical Chemistry, University of Belgrade, Studentski Trg 12-16, 11158 Belgrade, Serbia; gordana@ffh.bg.ac.rs; 5 Institute of General and Physical Chemistry, Studentski Trg 12-16, 11158 Belgrade, Serbia; zsaponjic@iofh.bg.ac.rs

**Keywords:** polyaniline, reduced graphene oxide, silver nanowires, hybrid nanocomposite, polycaprolactone, electromagnetic interference shielding, flexible films

## Abstract

The present study explores the comparative influence of reduced graphene oxide (rGO), silver nanowires (AgNWs), and their hybrid rGO–AgNWs on the electromagnetic interference (EMI) shielding performance of polyaniline (PANI)-based flexible films prepared using a polycaprolactone (PCL) matrix. The nanocomposites were synthesized through in situ oxidative polymerization of aniline in the presence of individual or hybrid fillers, followed by their dispersion in the PCL matrix and casting of the corresponding films. Morphological and structural characterization (SEM, Raman, and FTIR spectroscopy) confirmed a uniform PANI coating on both rGO sheets and AgNWs, forming hierarchical 3D conductive networks. Thermal (TGA) and thermomechanical (TMA) analyses revealed enhanced thermal stability and stiffness across all composite systems, driven by strong interfacial interactions and restricted polymer chain mobility. Tmax increased from 437.9 °C for neat PCL to 487.9 °C for PANI/PCL, 480.6 °C for PANI/rGO/PCL, 499.4 °C for PANI/AgNWs/PCL and 495.0 °C for the hybrid PANI/rGO–AgNWs/PCL film. The gradual decrease in contact angle following the order PANI/AgNWs/PCL < PANI/rGO–AgNWs/PCL < PANI/rGO/PCL < PANI/PCL < PCL clearly indicates a systematic increase in surface polarity and surface energy with the incorporation of conductive nanofillers. Electrical conductivity reached 60.8 S cm^−1^ for PANI/rGO/PCL, gradually decreasing to 27.4 S cm^−1^ for PANI/AgNWs/PCL and 22.1 S cm^−1^ for the quaternary hybrid film. The EMI shielding effectiveness (SET) measurements in the X-band (8–12 GHz) demonstrated that the PANI/rGO/PCL film exhibited the highest attenuation (~7.2 dB). In contrast, the incorporation of AgNWs partially disrupted the conductive network, reducing SE to ~5–6 dB. The findings highlight the distinct and synergistic roles of 1D and 2D fillers in modulating the electrical, thermal, and mechanical properties of biodegradable polymer films, offering a sustainable route toward lightweight, flexible EMI shielding materials.

## 1. Introduction

Electromagnetic interference (EMI) has become a major challenge in modern electronics, where closely integrated wireless and high-speed devices can unintentionally disrupt each other’s signals [1]. The growth of communication systems, radars, and IoT technologies has created a congested electromagnetic environment, leading to signal degradation, data loss, and potential health issues from long-term exposure [2]. Therefore, effective EMI shielding is crucial to ensure reliable device operation and user safety. Traditionally, metal enclosures or coatings have been used for their high conductivity and ability to reflect or absorb electromagnetic waves [3]. However, metallic shields are heavy, rigid, and prone to corrosion, prompting the development of lightweight, corrosion-resistant, and flexible alternatives.

Polymer-based EMI shielding composites have become promising options. These materials include a non-conductive polymer matrix loaded with conductive or magnetic fillers that reduce the transmission of electromagnetic waves [4]. Such systems offer low weight, corrosion resistance, and ease of processing, along with tunable electrical and magnetic properties. Common fillers include carbon nanotubes, graphene, metallic nanoparticles, or intrinsically conductive polymers [1]. When the filler content exceeds the percolation threshold, a connected conductive network forms, providing the composite with high conductivity and effective EMI shielding properties similar to those of metals [5].

Poly(ε-caprolactone) (PCL) has recently gained popularity as a biodegradable, flexible polymer matrix for electromagnetic interference (EMI) shielding composites. This semi-crystalline polyester, which melts at around 60 °C, offers excellent processability and mechanical flexibility. Using PCL supports the growing demand for sustainable electronic materials, as discarded devices often contribute to non-biodegradable waste. For example, PCL/MWCNT composites achieved about 30 dB of shielding at a 3 wt% loading while remaining biodegradable [6]. These findings demonstrate that environmentally friendly polymer composites can meet industrial EMI standards.

Polyaniline (PANI), an inherently conductive polymer, is added to improve both conductivity and dielectric loss [7]. It is lightweight, easy to process, and environmentally stable, with its conductivity adjustable through protonic doping. PANI also enhances toughness and flexibility when mixed with thermoplastics like PCL. From an electromagnetic perspective, PANI induces strong dielectric polarization and energy dissipation, leading to absorption-based shielding—preferred over reflection to minimize secondary EMI [8].

In this study, we developed a hybrid filler system consisting of reduced graphene oxide (rGO), silver nanowires (AgNWs), and polyaniline (PANI), dispersed in a PCL matrix and cast into flexible films. Each filler component provides specific electrical and dielectric properties necessary for EMI attenuation. rGO, a reduced form of graphene oxide, shows high electrical conductivity, a large surface area, and residual oxygen groups that promote interfacial polarization [9]. Even at low loadings, it forms extensive percolating networks that enhance electrical conductivity and absorption. AgNWs, with an ultra-high aspect ratio and metallic conductivity, complement rGO by offering one-dimensional conduction pathways. Silver’s high intrinsic conductivity and resistance to oxidation make it suitable for EMI reflection [10]. For example, Liu et al. demonstrated flexible textiles coated with AgNWs with approximately 59 dB shielding effectiveness across 5–18 GHz at only 1.4 mm thickness [11]. Within the PCL matrix, AgNWs are expected to connect rGO nanosheets, improving charge transport and the conductivity of the network. PANI coatings on rGO and AgNWs may improve filler dispersion and interfacial adhesion, potentially resulting in more uniform and efficient composites.

The combination of rGO, AgNWs, and PANI is expected to produce synergistic effects that exceed those of any individual filler. rGO forms extensive 2D conductive frameworks, AgNWs act as metallic bridges for long-range charge transport, and PANI provides dielectric absorption and interface compatibility. Together, they can likely form a hierarchical 3D network capable of effective EMI attenuation at low filler content.

Literature supports such carbon–polymer and metal–polymer systems, which frequently achieve improved shielding effectiveness and mechanical flexibility [8,12,13,14,15,16,17]. For instance, Das et al. demonstrated that PANI–PS–GO thin films reached an EMI shielding effectiveness of about 40 dB in the X-band (8–12 GHz) at low GO loadings [12]. Similarly, Nasir et al. synthesized PANI-grafted GO nanohybrids and reported enhanced electrical conductivity and interfacial interactions, highlighting their potential for EMI and electronic applications [13]. Reduced graphene oxide (rGO)/polyaniline (PANI) hybrids show great promise as lightweight, flexible microwave-absorbing, and EMI-shielding materials due to their synergistic dielectric properties and improved interfacial polarization [15]. Incorporating PANI and rGO into paraffin wax significantly improves mechanical strength, electrical conductivity, and dielectric response, indicating the suitability of these nanocomposites for effective EMI shielding [15]. In situ synthesized PANI/S-RGO nanocomposites demonstrate greatly increased thermal stability and EMI shielding effectiveness, achieving an absorption-dominant mechanism with a total SE above 20 dB across 1–20 GHz [8]. Fang et al. prepared layer-structured AgNWs/PANI composite films containing approximately 14 vol% AgNWs and achieved conductivities around 5300 S cm^−1^, with EMI shielding exceeding 50 dB in the X-band [14].

Although research specifically on PANI/GO/AgNWs composites is limited, related studies indicate that these combinations can create lightweight, flexible, and high-performance EMI shields [17].

To the best of our knowledge, this is the first report describing the fabrication of lightweight, flexible PANI/rGO/AgNWs nanocomposite films within a PCL matrix, demonstrating remarkable EMI shielding efficiency and potential for advanced flexible electronics.

## 2. Results

### 2.1. Surface Morphology of Synthesized Materials

**Figure 1:**
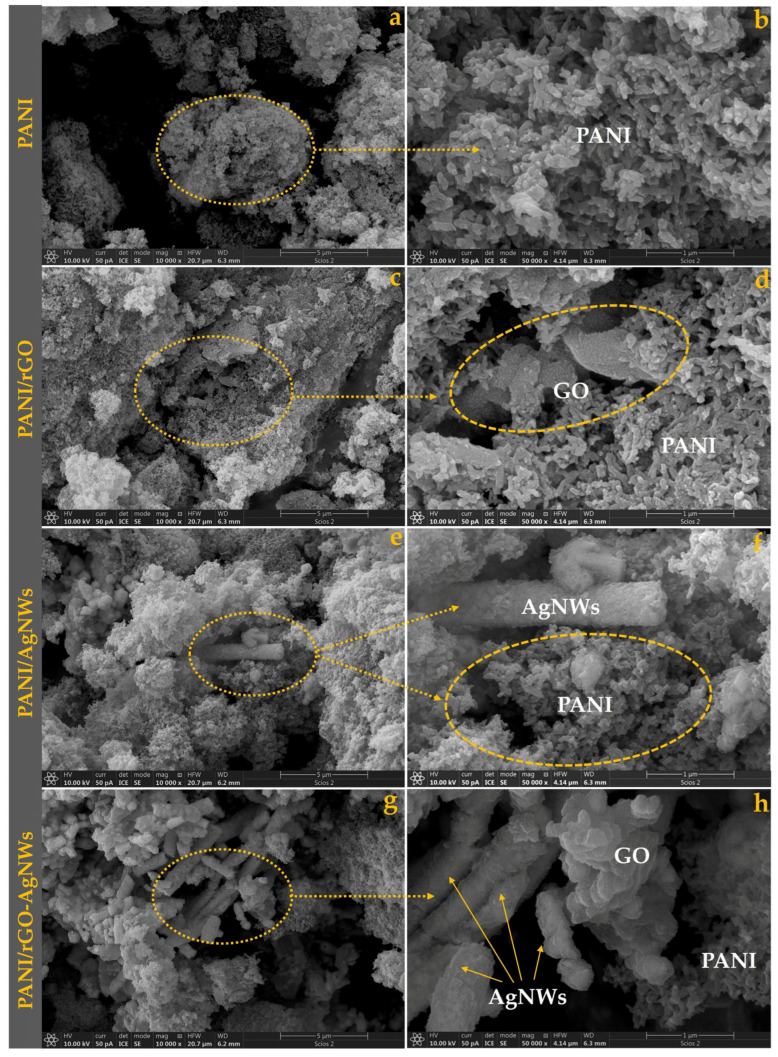
SEM micrographs of pristine PANI (**a**,**b**) and its nanocomposites with: rGO (**c**,**d**), AgNWs (**e**,**f**), and the rGO–AgNWs hybrid (**g**,**h**).

To examine the morphology and surface characteristics of the prepared powdered materials, scanning electron microscopy (SEM) was employed. SEM images of all the synthesized samples are shown in Figure 1.

The morphology of pristine PANI (Figure 1a,b) reveals an aggregated, granular structure composed of irregularly shaped nanofibrous particles that form a porous network [18]. The surface appears rough and uneven, consisting of primary grains with a diameter of approximately 50–100 nm that cluster into micron-sized aggregates (around 0.5 to 1 µm). This porous, interconnected structure is typical of PANI produced by oxidative polymerization, allowing efficient charge transport through numerous interparticle contacts [19,20].

In the PANI/rGO composite, rGO nanosheets (0.5–2 µm lateral size) are partially coated with PANI grains, indicating uniform polymer deposition on the graphene surface (Figure 1c,d). The wrinkled, layered structure of rGO remains visible, creating conductive bridges between polymer cluster islands. The presence of these extended 2D sheets introduces a flaky–granular hybrid morphology, with increased surface roughness and interfacial contact [21]. The firm wrapping of PANI around rGO suggests strong π–π interactions and effective charge transfer pathways, which are expected to improve electrical conductivity and EMI shielding efficiency [22].

Adding silver nanowires creates a distinct fibrous–granular structure (Figure 1e,f). The AgNWs, clearly visible as elongated filaments measuring 50–100 nm in diameter and 5–20 µm in length, are embedded within the PANI matrix, forming conductive bridges between polymer domains. The PANI coating partially covers the nanowires, ensuring close interfacial contact. This one-dimensional network enhances overall electrical percolation, facilitating electron transport through the polymer [23]. The presence of granular PANI alongside metallic nanowires results in a denser, more interconnected microstructure compared to pure PANI. These structural features are known to significantly boost electrical conductivity and, consequently, the EMI shielding performance of polymer-metal hybrid systems [24].

The ternary hybrid exhibits the most complex, hierarchically organized morphology (Figure 1g,h). The micrographs show rGO sheets decorated with AgNWs, which are coated and interconnected by PANI granules. This configuration creates a 3D conductive framework where the 2D (rGO) and 1D (AgNWs) fillers are closely integrated and bridged by the conductive polymer. The AgNWs, randomly oriented yet evenly distributed, are effectively anchored on the rGO surfaces, preventing aggregation and enhancing filler dispersion. The resulting morphology provides multiple electron pathways, strong interfacial polarization sites, and high structural stability, all of which work together to improve EMI shielding performance.

**Figure 2:**
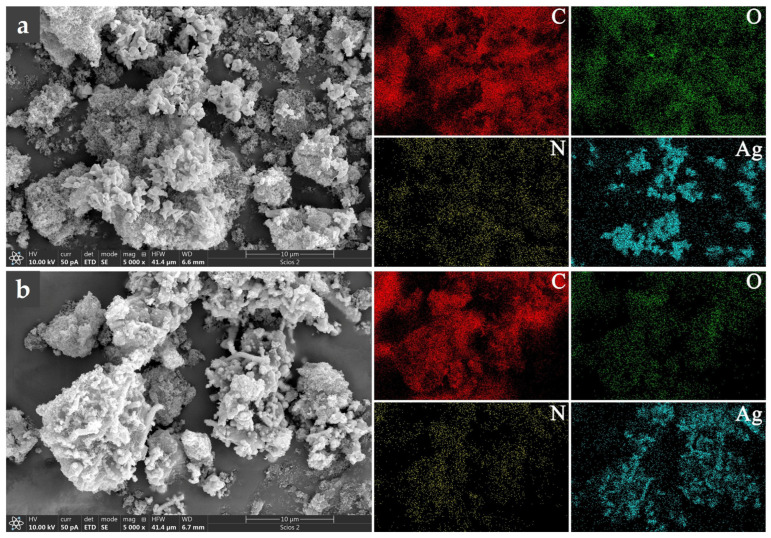
EDS elemental mapping for PANI/AgNWs (**a**), and PANI/rGO-AgNWs (**b**). Elemental distribution is shown using different colors: red for carbon, green for oxygen, yellow for nitrogen, and cyan for silver.

To evaluate the uniformity of the polyaniline coating and the distribution of the constituents within the nanocomposites, EDS elemental mapping was performed on representative PANI/AgNWs and PANI/rGO–AgNWs samples. These two composites were chosen as representative systems because they exhibit different filler architectures—one containing only AgNWs and the other including both rGO and AgNWs—allowing a more precise assessment of how hybrid fillers affect coating morphology and elemental distribution. The corresponding results are shown in Figure 2.

The EDS elemental maps confirm the presence and distribution of carbon (C), oxygen (O), nitrogen (N), and silver (Ag) within the analyzed composites. The nitrogen signal, originating solely from polyaniline, is uniformly distributed across the entire mapped area of both samples, indicating the successful formation of a continuous PANI coating over the conductive filler network.

In the case of the PANI/AgNWs composite (Figure 2a), the Ag signal appears as distinct, localized regions corresponding to AgNWs, revealing partially aggregated nanowire domains embedded within the PANI matrix. In contrast, the PANI/rGO–AgNWs composite (Figure 2b) shows a more complex and interconnected morphology. The broader Ag-rich regions observed in the EDS maps indicate partial aggregation of AgNWs, likely promoted by their interaction with rGO sheets during composite formation. At the same time, the uniform nitrogen distribution further confirms the presence of a continuous PANI coating enveloping the hybrid filler network.

### 2.2. Molecular Structure

**Figure 3:**
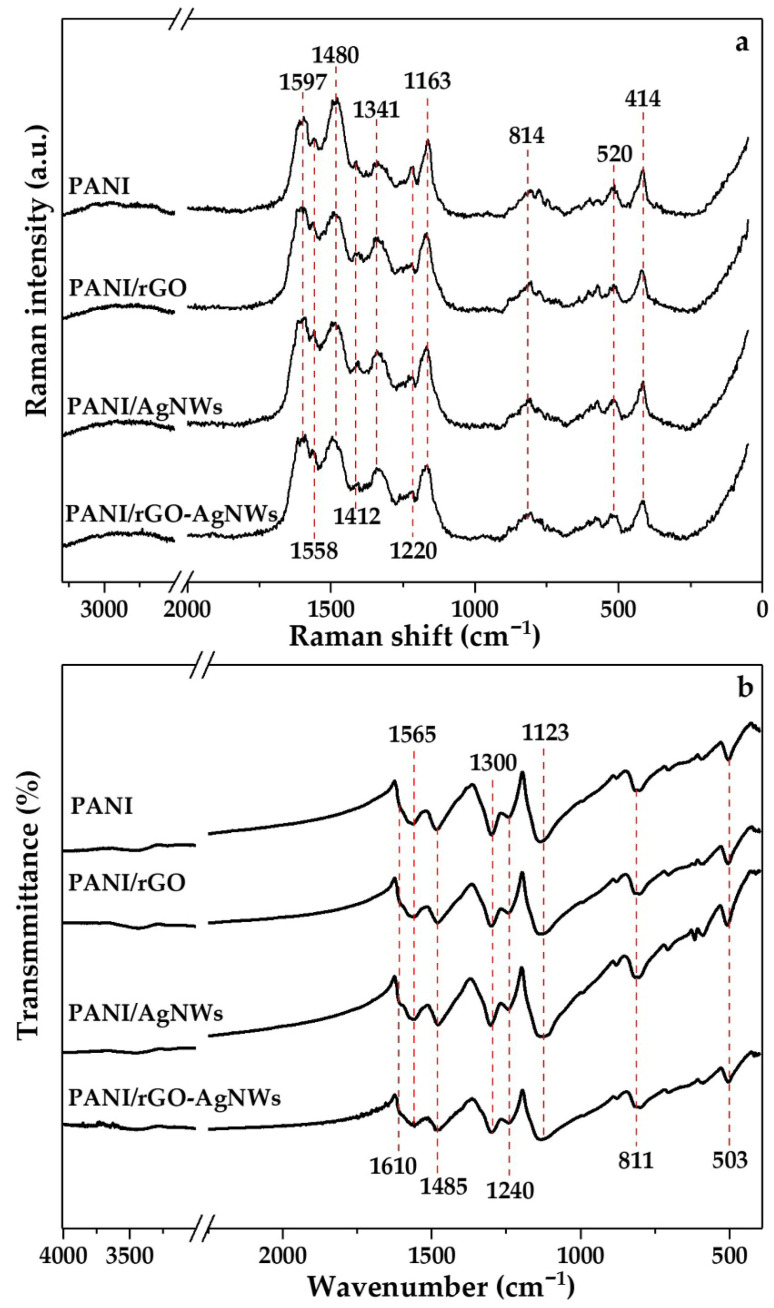
Raman (**a**) and FTIR (**b**) spectra of neat PANI and its nanocomposites: PANI/rGO, PANI/AgNWs, and PANI/rGO–AgNWs.

The molecular structures of the synthesized materials were analyzed using Raman and FTIR spectroscopies. The Raman and FTIR spectra of the synthesized materials are shown in Figure 3a and Figure 3b, respectively.

In the Raman spectrum of pristine PANI, characteristic vibrational features associated with its conducting emeraldine salt oxidation state are evident. One of the most prominent bands appears at 1597 cm^−1^, corresponding to C=C and C~C stretching vibrations of the quinonoid (Q) and semi-quinonoid (SQ) rings (where “~” indicates a bond between single and double). The band at 1558 cm^−1^ relates to the C=C stretching vibration of Q rings and can also be connected with phenazine units in PANI, along with the weak band at 1412 cm^−1^ [25]. The strongest band in the spectrum occurs at 1480 cm^−1^ and is attributed to C=N stretching vibrations in Q units [25,26,27]. The band at 1341 cm^−1^ is assigned to C~N•+ stretching vibration of the delocalized polaronic structure, indicating a conductive form of PANI [25]. The band at 1220 cm^−1^ corresponds to C–N stretching in benzenoid (B) units, and the peak at 1163 cm^−1^ relates to C–H in-plane bending vibrations of the SQ and Q rings [28,29,30]. Additionally, the signal at 814 cm^−1^ corresponds to out-of-plane C–H bending in para-disubstituted benzene rings, confirming the linear head-to-tail coupling of aniline units [31,32]. The low-frequency modes at 520 and 414 cm^−1^ are associated with deformation vibrations of the aromatic skeleton and N–H wagging, which, although less intense, are consistently observed in conducting PANI [33].

The incorporation of nanostructured components results in slight spectral changes, suggesting possible interfacial interactions and alterations to the electronic structure of PANI. In the Raman spectra of PANI/rGO, PANI/AgNWs, and PANI/rGO–AgNWs composites, the polaron band at 1341 cm^−1^ shows an increase in the intensity (e.g., related to the band at 1480 cm^−1^) compared to that band in the spectrum of pristine PANI, reflecting enhanced charge delocalization and stronger interfacial interactions between PANI chains and the conductive nanofillers. Blue-shifts are noted for the band at 1163 cm^−1^, which moves to wavenumbers 1173, 1167, and 1171 cm^−1^ for the PANI/rGO, PANI/AgNWs, and PANI/rGO–AgNWs samples, respectively, indicating formation of larger amounts of polarons (SQ rings) and increase in PANI conductivity, due to interchain conductive ‘bridges’ formed by rGO and AgNWs. Additionally, all composite spectra show a reduction in the intensity of the bands at 1480 and 1220 cm^−1^ compared to pristine PANI, attributed to transformation of Q to SQ rings, changes in the conjugation length and partial disruption of benzenoid–quinonoid structures caused by strong interfacial interactions between PANI chains and the nanofillers [34,35].

The simultaneous shifts in peak positions, changes in relative intensities, and the appearance of new features collectively confirm that the hybridization of PANI with rGO and AgNWs promotes strong electronic coupling and structural reorganization within the polymer chains. Such modifications not only stabilize the conductive emeraldine salt form but also enhance the density and mobility of charge carriers (polarons and bipolarons). These findings are consistent with previous Raman studies of PANI-based composites [25], which consistently highlight the sensitivity of the Raman response to doping, oxidation state, and interfacial interactions.

The FTIR spectrum of pristine PANI displays a series of absorption bands characteristic of its emeraldine oxidation state. The two prominent bands at 1565 cm^−1^ and 1485 cm^−1^ correspond to the C=C stretching vibrations of Q and B rings, respectively [26,36,37,38]. The absorption band at 1300 cm^−1^ is assigned to C–N stretching of secondary aromatic amines, while the band around 1240 cm^−1^ relates to C–N•+ stretching vibrations associated with charged structures of the polaronic form of emeraldine salt [26,39,40]. The latter serves as a diagnostic marker of doping, directly linked to the formation of polaronic charge carriers. Another characteristic feature of doped PANI is the broad band at 1123 cm^−1^, often called the “electronic-like band,” originating from the stretching vibrations of –NH^+^= groups and the vibrations of charged units Q=NH+−B or B−NH+•−B, coupled with delocalized polaronic states along the polymer backbone [41,42,43]. Its strong intensity indicates effective protonation, extended conjugation, and good conductivity [19,20,21].

Furthermore, the band at 811 cm^−1^ is the dominant feature in the ‘substitution pattern’ 900–650 cm^−1^ region and results from out-of-plane C–H bending vibrations in para-disubstituted benzene rings, confirming the prevalent head-to-tail coupling of aniline units during oxidative polymerization, which leads to regular, linear PANI chains [26,44,45]. The low-frequency feature at 503 cm^−1^ is attributed to skeletal ring deformations and C-H out-of-plane bending vibrations. It is generally recognized as a subtle but consistent marker of the PANI framework [46].

When nanostructured fillers are incorporated, minor band shifts are observed. Compared to pristine PANI, the spectra of PANI/rGO, PANI/AgNWs, and PANI/rGO–AgNWs show slight shifts in the bands from 1300 to 1303, 1304, and 1302 cm^−1^, and from 1485 to 1480, 1478, and 1477 cm^−1^, respectively. These minor spectral changes are likely due to interactions between the π-electron systems of PANI and the conductive nanofillers such as rGO and AgNWs.

### 2.3. Thermogravimetric Analysis

Thermogravimetric (TGA) and differential thermogravimetric (DTG) analyses were performed to evaluate the thermal stability and decomposition behavior of the prepared powder and film samples.

#### 2.3.1. Thermal Properties of Powder Samples

**Figure 4:**
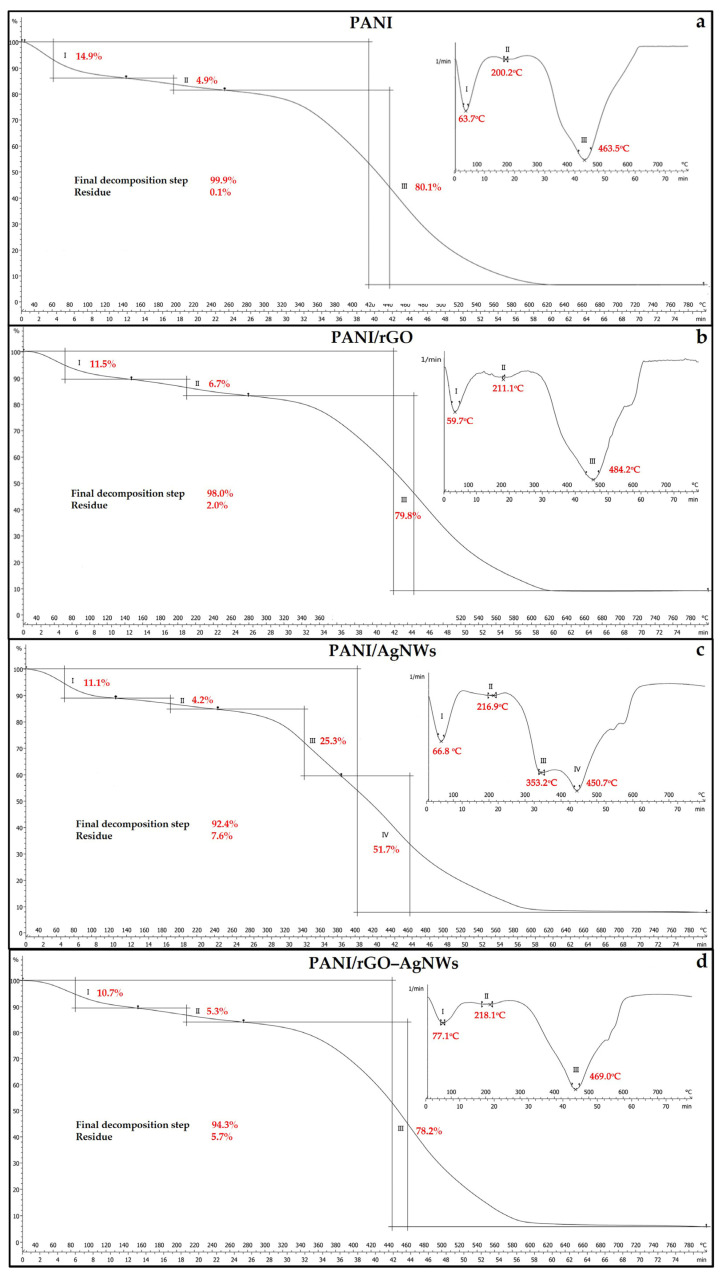
Thermogravimetric analysis of synthesized powder samples: PANI (**a**), PANI/rGO (**b**), PANI/AgNWs (**c**), and PANI/rGO–AgNWs (**d**).

The TGA/DTG curves of pure PANI, PANI/rGO, PANI/AgNWs, and PANI/rGO-AgNWs nanocomposite powder samples are shown in Figure 4.

Pure PANI undergoes three main stages of degradation (Figure 4a). The first stage involves a 14.9% mass loss at a DTG peak of 63.7 °C, which indicates the removal of physically adsorbed moisture [47]. In the second stage, about 4.9% of the mass is lost at a DTG peak of 200.2 °C, due to the elimination of dopant molecules (HCl, H_2_SO_4_) and low-molecular-weight fragments [48,49]. The most significant weight loss, 80.1%, occurs between 300 and 500 °C, with a DTG peak at 463.5 °C, attributed to the breakdown of the primary polymer backbone and the oxidative degradation of the carbonaceous residue [47]. Beyond 600 °C, the curve stabilizes as the PANI sample is fully degraded.

The addition of rGO enhances the thermal stability of PANI (PANI/rGO), moving the main degradation point to a higher temperature (484.2 °C) compared to pure PANI (Figure 4b). The DTG curve displays three distinct peaks at 59.7 °C, 211.1 °C, and 484.2 °C, corresponding to water release, PANI dopant removal, and polymer chain breakdown, respectively. These degradation stages are associated with weight losses of 11.5%, 6.7%, and 79.8%, respectively. The residual mass is around 2.0%. The presence of rGO sheets hinders the diffusion of volatile decomposition products and acts as a physical barrier to heat transfer, thereby enhancing the composite’s thermal stability [50].

The TGA/DTG curves of the PANI/AgNWs sample show four distinct degradation regions (Figure 4c). The initial weight loss of 11.1% at the DTG peak of 66.8 °C results from moisture evaporation, followed by a minor loss (4.2%) near 216.9 °C due to dopant removal and structural reorganizations. The primary degradation steps occur at 353.2 °C (25.3%), associated with breaking of PANI chains, and at 450.7 °C (51.7%), indicating the final oxidation of the remaining carbonaceous material. After degradation, a residual char of 7.6% was observed, suggesting the presence of an inorganic component (AgNWs). A noticeable shift to higher decomposition temperatures than for pure PANI is observed in the second degradation step, while the main degradation stage splits into two broader steps.

Among all tested samples, the PANI/rGO-AgNWs hybrid exhibits the highest thermal stability (Figure 4d). The DTG peaks appear at 77.1 °C, 218.1 °C, and 469.0 °C, indicating a slower and more controlled degradation process. The related weight losses are 10.7%, 5.3%, and 78.2%, respectively. The strong π–π interactions between PANI and rGO, along with the uniform distribution of AgNWs, form a compact and cross-linked conductive network that effectively slows thermal decomposition [51]. The synergistic effect of rGO and AgNWs improves the thermal stability of the PANI/rGO-AgNWs hybrid by enhancing stability at all three degradation steps, resulting in a residue of 5.7% [52].

#### 2.3.2. Thermal Properties of Film Samples

**Figure 5:**
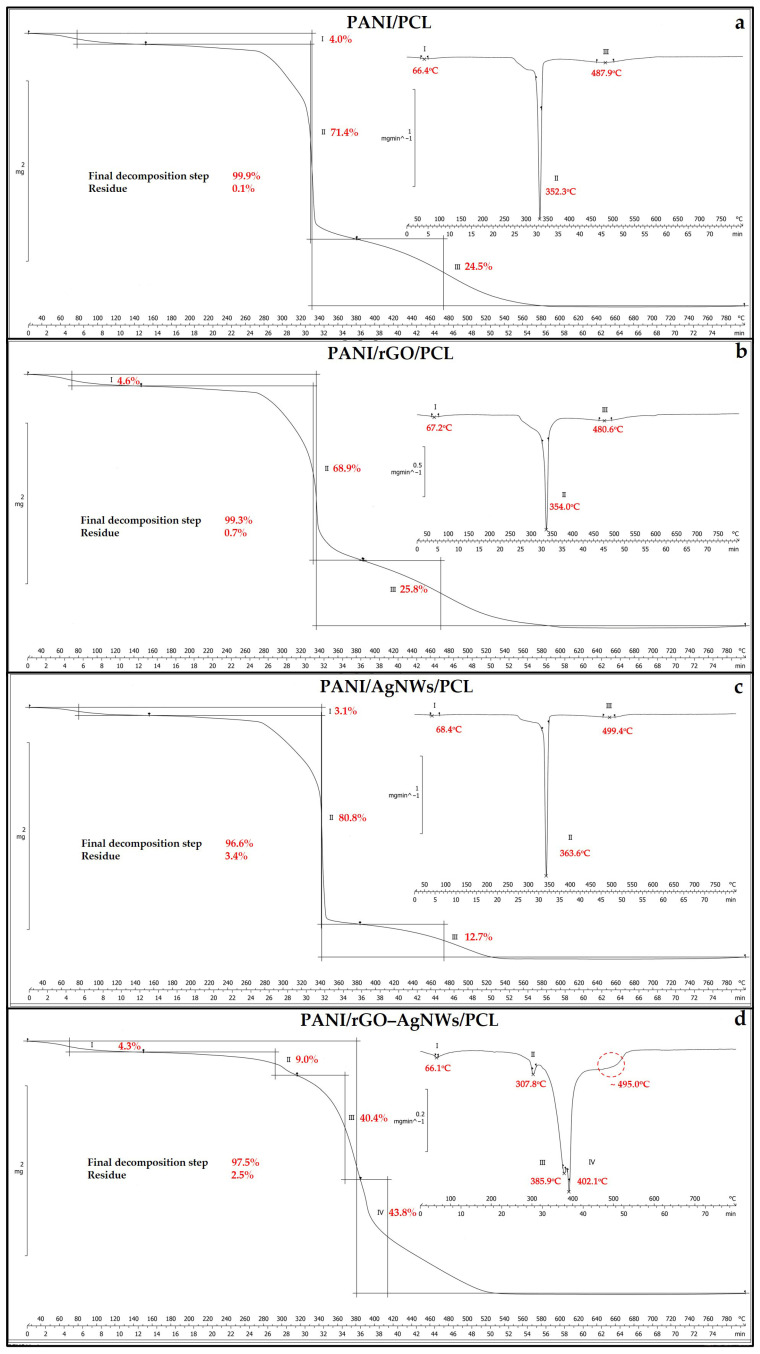
Thermogravimetric analysis of synthesized film samples: PCL (**a**), PANI/PCL (**b**), PANI/rGO/PCL (**c**), PANI/AgNWs/PCL (**c**), and PANI/rGO–AgNWs/PCL (**d**).

The thermogravimetric analysis (TGA) and derivative thermogravimetry (DTG) curves of pristine PCL, PANI/PCL, and the nanocomposite PCL-casted films with rGO, AgNWs, and rGO–AgNWs hybrid fillers are shown in Figure 5. The results offer insights into the thermal decomposition behavior and how each component affects the overall stability of the hybrid systems. The curves display the typical multistep decomposition patterns associated with polymer chain scission, oxidative degradation, and the subsequent burning of the carbon-rich residue.

For pristine PCL (Supplementary Materials, Figure S1), a significant mass-loss step (≈81.7%) was observed between 240 °C and 370 °C, with a DTG peak at 314.9 °C. This was accompanied by two smaller degradation steps, with DTG peaks at 395.1 °C and 437.9 °C, corresponding to the thermo-oxidative degradation of the aliphatic polyester backbone and the volatilization of low-molecular-weight fragments and combustion of residual organic material, respectively. The minimal residue (0.01%) indicates complete oxidation of organic components under the tested conditions, consistent with the known behavior of PCL in oxygenated environments.

When PANI is incorporated into the PCL matrix, the PANI/PCL composite (Figure 5a) exhibits a 71.4% mass loss during the main degradation stage, with a DTG peak at 352.3 °C, indicating oxidative scission of the PCL matrix. This is followed by the decomposition of doped PANI chains (24.5%, 487.9 °C) [47,53]. The initial low-temperature shoulder near 66 °C is attributed to the evaporation of residual moisture and dopant species [47].

In the PANI/rGO/PCL system (Figure 5b), the main degradation steps shifted to slightly higher temperatures (354.0 °C → 480.6 °C) indicating a stabilizing effect of reduced graphene oxide. The strong interfacial interactions between rGO nanosheets and PANI chains can inhibit oxygen diffusion and prevent chain scission reactions, thereby delaying oxidation [54]. The improved thermal stability supports the development of a partial barrier structure that reduces heat transfer and oxygen penetration within the composite matrix.

An additional improvement was observed in the PANI/AgNWs/PCL film (Figure 5c), where decomposition occurred at 363.6 °C and 499.4 °C. The silver nanowires likely help dissipate heat and promote partial crosslinking during heating, leading to the formation of thermally stable oxidized fragments. Metallic nanostructures are known to act as heat sinks, thereby increasing the thermal stability of conductive polymer composites [55].

The quaternary PANI/rGO–AgNWs/PCL system (Figure 5d) exhibited the most complex decomposition profile, with four distinct stages. The initial minor event at 66 °C (≈4.3%) is due to moisture release; the second at 307.8 °C (≈9.0%) corresponds to the oxidation of low-molecular-weight segments; the third (≈40.4%) and fourth (≈43.8%) stages at 385.9 °C and 402.1 °C represent overlapping degradation of the PANI backbone and the PCL matrix. The final residue increased to 2.5%, indicating the synergistic stabilization provided by the dual rGO–AgNWs hybrid network [47,53]. The conductive framework serves as both a physical barrier and a heat-dissipation pathway, slowing oxidative decomposition and promoting char formation.

Quantitative TGA/DTG parameters derived from Figure 4 are summarized in Table 1. Compared to pure PCL (Tmax = 437.9 °C; negligible residue), the addition of conductive fillers shifts the main decomposition to higher temperatures and increases char yield. In the case of PANI/PCL, Tmax increases significantly to 487.9 °C, while Tmax for PANI/rGO/PCL reaches 480.6 °C. PANI/AgNWs/PCL attains the highest Tmax (499.4 °C) and has a char residue of 3.4%, which aligns with the heat-dissipation function of AgNWs and their contribution to thermally stable oxidized fragments. The quaternary PANI/rGO–AgNWs/PCL exhibits a four-step profile (Tmax ≈ 495.0 °C) with a higher residue (2.5%), attributed to the synergistic barrier effect and partial heat sinking within the hybrid 1D/2D network. Overall, the trends in Tonset, Tmax, and residues confirm that rGO and AgNWs act as effective thermal stabilizers by limiting chain mobility, hindering oxygen diffusion, and promoting char formation.

The TGA results show that both rGO and AgNWs serve as effective thermal stabilizers by enhancing interfacial interactions, limiting chain mobility, and improving heat conduction within the hybrid polymer network.

### 2.4. Thermomechanical Analysis

**Figure 6:**
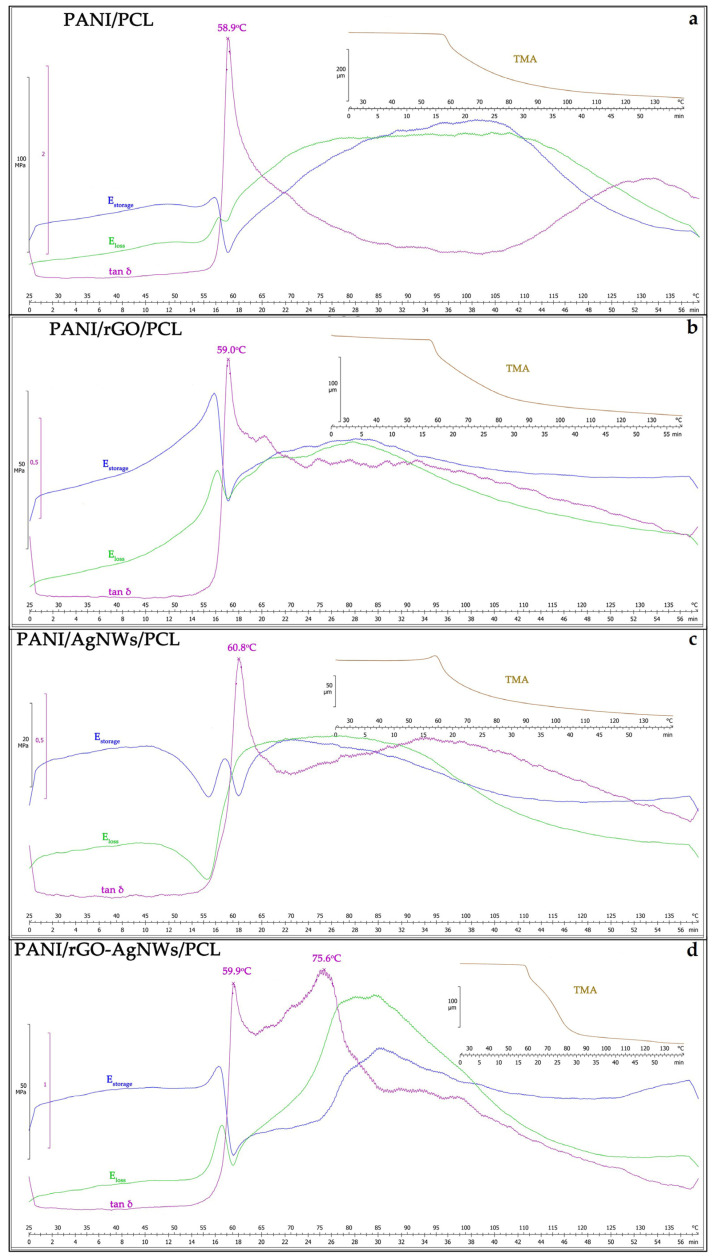
Thermomechanical analysis of synthesized film samples: PANI/PCL (**a**), PANI/rGO/PCL (**b**), PANI/AgNWs/PCL (**c**), and PANI/rGO–AgNWs/PCL (**d**).

The thermomechanical behaviour of the PANI/PCL-based composite films was examined to evaluate the impact of conductive fillers (rGO and AgNWs) on the viscoelastic and dimensional stability of the hybrid systems (Figure 6). The TMA and DMA curves (E′, E″, and tan δ) reveal distinct transitions related to the softening and segmental mobility of the PCL phase, as well as the reinforcing effect of PANI and the incorporated nanofillers [56,57].

For the pristine PANI/PCL sample (Figure 6a), a single relaxation peak was observed in the tan δ curve at 58.9 °C, corresponding to the glass transition of the PCL phase. The associated decrease in storage modulus (E′) and the increase in loss modulus (E″) indicate the onset of polymer-chain relaxation and reduced stiffness above this temperature, which is typical for semi-crystalline polyesters such as PCL [58]. The TMA curve shows a pronounced dimensional change, confirming the matrix softening in this region.

The incorporation of rGO nanosheets caused a slight shift in the tan δ maximum to 59.0 °C (Figure 6b), indicating minor restriction of polymer-chain mobility due to interfacial π–π and hydrogen-bonding interactions between rGO and the PCL–PANI matrix [59,60]. The reduced amplitude of the tan δ peak also suggests enhanced energy dissipation control and better stiffness retention. This behaviour aligns with improved interfacial adhesion and physical crosslinking induced by the planar structure of rGO, as previously observed in PCL/GNP and GO/epoxy systems, where strong filler–matrix adhesion leads to increased E′ and a narrowing of tan δ [61,62].

A more pronounced reinforcement was observed after adding AgNWs (Figure 6c). The tan δ maximum shifted to 60.8 °C, along with a significant increase in storage modulus and a reduction in the loss-modulus peak. This shows that the silver nanowires effectively limit polymer chain movement and improve the thermal and dimensional stability of the PANI/AgNWs/PCL film. Similar patterns—a higher Tg and an increase in E′ because of forming a continuous nanowire network—have been reported for AgNWs/epoxy nanocomposites [63,64]. The TMA profile supports this, showing the smallest dimensional expansion among all binary systems, which indicates a denser, more cohesive structure.

The ternary PANI/rGO–AgNWs/PCL composite (Figure 6d) showed two relaxation events, with the first peak at 59.9 °C and a secondary transition at 75.6 °C. The presence of the second relaxation indicates a synergistic effect between the rGO and AgNWs fillers, leading to heterogeneous interfacial regions with different mobility. Similar dual-relaxation behavior has been observed in polymer nanocomposites containing both 1D and 2D fillers, where the hybrid network forms domains with varying degrees of chain constraint [65]. The broadening and upward shift in the relaxation peaks, along with the higher E′ values, confirm that the combined rGO and AgNWs network creates a highly interconnected structure within the PANI/PCL matrix, capable of efficiently transferring stress and limiting molecular motion at higher temperatures [66].

Overall, the TMA and DMA results demonstrate a clear trend of increasing thermomechanical stability from pristine PANI/PCL to the quaternary PANI/rGO–AgNWs/PCL system. The improved behavior results from strong filler–matrix interfacial interactions, uniform nanofiller dispersion, and the formation of continuous conductive networks that serve as physical crosslinking points [67]. These findings support previous reports of synergistic effects in hybrid 1D/2D nanostructures, which offer superior mechanical strength, heat resistance, and energy-damping capabilities compared to single-filler composites [68]. Such enhancements confirm the suitability of the PANI/rGO–AgNWs/PCL films for flexible electronic and EMI-shielding applications, where maintaining mechanical integrity under thermal stress is essential.

### 2.5. Contact Angle Measurements

**Figure 7:**
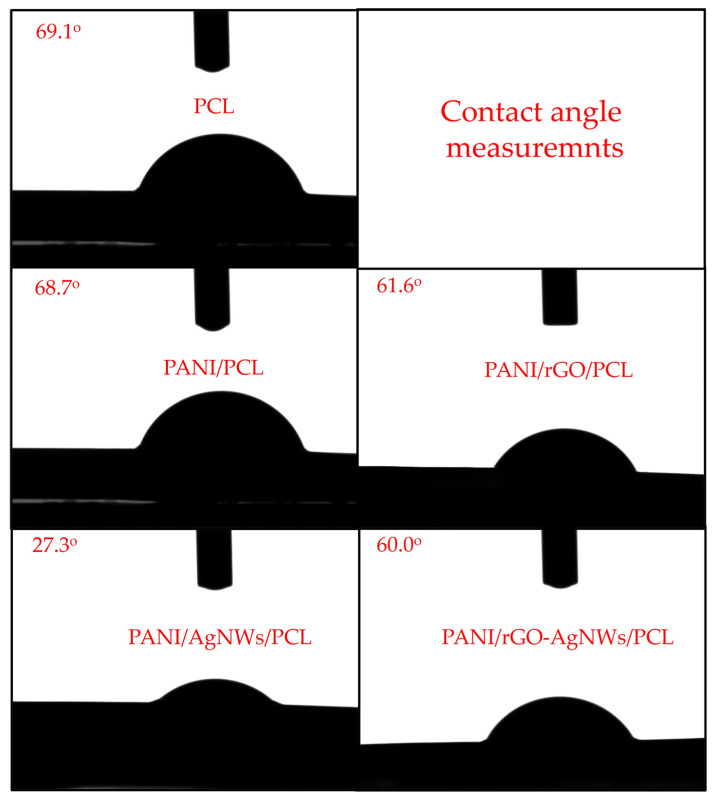
Contact angle of neat PCL film, and synthesized composite films: PANI/PCL, PANI/rGO/PCL, PANI/AgNWs/PCL, and PANI/rGO–AgNWs/PCL.

The contact angle measurements revealed distinct differences in the wettability of the tested films, highlighting the influence of surface chemistry, morphology, and filler interactions. The results are shown in Figure 7.

The smooth PCL film exhibited a contact angle of ~69.1°, indicating its moderately hydrophobic aliphatic structure. The preparation method and material thickness significantly impact the contact angle of PCL. Additionally, this value can vary depending on the molecular weight of PCL and the solvent used. For the method employed in this study (solution-cast PCL film on glass), the reported contact angle typically ranges from 65° to 85°, consistent with our findings [69,70,71].

The PANI/PCL film displayed a slightly lower contact angle (~68.7°) due to the presence of polar amine and imine groups that can form hydrogen bonds with water molecules, thereby slightly increasing surface polarity. Similar results are observed in the literature [72].

Incorporating reduced graphene oxide (rGO) into the composite lowered the contact angle to about 61.6°, indicating improved wettability. This enhancement is due to oxygenated functional groups remaining on the rGO surface, which create additional polar sites capable of hydrogen bonding with water molecules. Subtil et al. examined how rGO concentration affected the contact angle of polyethersulfone/rGO/PANI composites, with results ranging from 45° to 54°, depending on the amount of rGO used and the dopant molecule [73].

The PANI/AgNWs/PCL film exhibited the lowest contact angle (27.3°), indicating a highly hydrophilic surface. Similar results are seen with the PANI/AgNWs membrane, where the contact angle decreases to approximately 40° [74]. This significant reduction suggests a substantial increase in surface energy, likely because AgNWs facilitate local charge buildup and capillary effects that enhance water spreading.

Interestingly, the quaternary PANI/rGO–AgNWs/PCL composite showed an intermediate contact angle (~60°). This indicates a balance between rGO-induced roughness and AgNWs-induced polarity on one side, and the partially hydrophobic PANI/PCL matrix on the other.

From a functional perspective, the observed trend—PANI/AgNWs/PCL < PANI/rGO–AgNWs/PCL < PANI/rGO/PCL < PANI/PCL < PCL—shows a systematic change in surface polarity and energy as conductive nanofillers are added.

### 2.6. EMI Shielding and Conductivity Measurements

**Figure 8:**
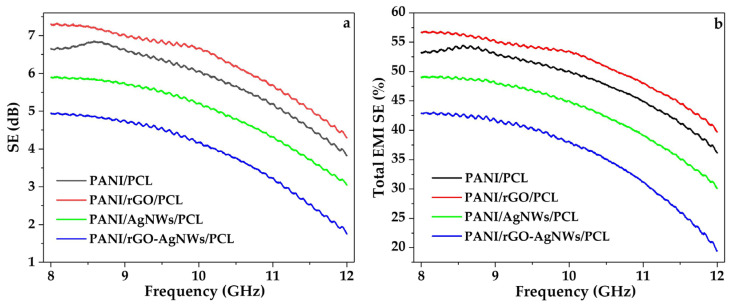
Figure X. EMI shielding performance of PANI-based composite films in the X-band (8–12 GHz): (**a**) Shielding effectiveness (SE), (**b**) Corresponding total EMI SE (%) for PANI/PCL, PANI/rGO/PCL, PANI/AgNWs/PCL, and PANI/rGO-AgNWs/PCL films.

The electromagnetic interference shielding effectiveness (EMI SE) of PANI/PCL, PANI/AgNWss/PCL, PANI/rGO/PCL, and PANI/rGO–AgNWs/PCL hybrid films was tested within the X-band frequency range (8–12 GHz), and the results are shown in Figure 8. It is clear that adding conductive nanofillers (rGO and AgNWs), whether alone or combined (rGO/AgNWs), significantly impacts the overall EMI shielding performance of the materials.

The highest total EMI SE (≈7.2 dB, Figure 8a) was observed in the PANI/rGO/PCL system, which blocked over 50% of incident electromagnetic radiation in the 8–12 GHz range (Figure 8b). This improvement demonstrates a strong interaction between PANI and rGO, promoting electron tunneling and the formation of extended conductive networks within the polymer matrix. Similar behavior has been reported in previous studies, where electron tunneling between rGO sheets and PANI was a key factor in EM wave absorption [13,75,76].

In contrast, the lowest SE was observed for the PANI/rGO–AgNWs/PCL hybrid composite. The addition of AgNWs seems to decrease the shielding efficiency in all the composites they are part of. This is due to partial aggregation of AgNWs (confirmed by SEM -EDS measurements), leading to inhomogeneity and affecting the conductive networks of rGO and PANI.

To further clarify the relationship between EMI SE and electrical conductivity, all synthesized films were tested for their electrical conductivity. The PANI/rGO/PCL film exhibited the highest conductivity at 60.8 S cm^−1^, which gradually decreased to 39.0 S cm^−1^ for PANI/PCL, 27.4 S cm^−1^ for PANI/AgNWs/PCL, and 22.1 S cm^−1^ for the ternary PANI/rGO–AgNWs/PCL nanocomposite film. These results align with the trend observed in EMI shielding measurements, emphasizing their close connection. This correlation indicates that enhanced charge transport within the composite network directly improves shielding efficiency. The importance of the conductivity mechanism in EMI shielding properties was comprehensively discussed in the study by Hou et al. [77].

Overall, the shielding performance of these composites arises from a synergistic interaction among conduction loss, interfacial polarization, and, eventually, multiple internal reflection processes. The two-dimensional rGO sheets effectively facilitate electron hopping and dipolar polarization, while one-dimensional AgNWs serve as metallic reflection centers. However, their combined inclusion in a single matrix does not produce a purely additive effect; instead, it creates a complex balance between maintaining conductive network connectivity and impedance matching. Therefore, PANI/rGO/PCL exhibits the highest EMI SE mainly due to dissipation mechanisms. PANI/AgNWs/PCL shows moderate performance, whereas PANI/rGO–AgNWs/PCL exhibits more complex behavior, influenced by partial network disruption. In both AgNWs-containing samples, nanowire aggregation (Figure 2) plays a crucial role, disrupting continuous conductive pathways and significantly impacting EMI shielding effectiveness (SE).

**Scheme 1:**
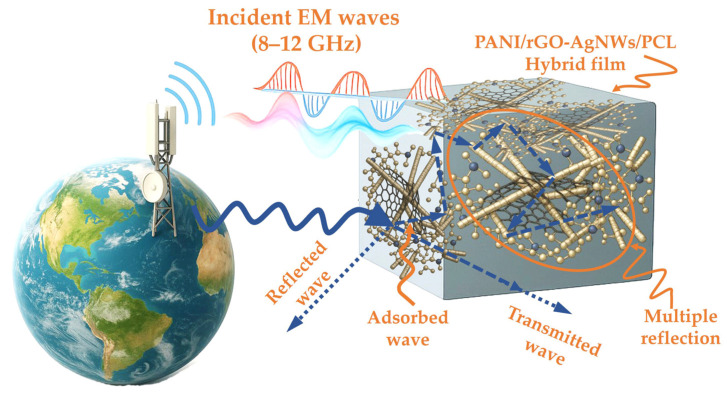
Graphical illustration of the shielding mechanism of the synthesized PANI/rGO–AgNW/PCL hybrid films.

Scheme 1 shows the possible EMI shielding mechanism of the PANI/rGO–AgNWs/PCL hybrid film. When exposed to electromagnetic waves (8–12 GHz), the hybrid structure supports diverse processes, including absorption, transmission, reflection, and multiple internal reflections within the conductive network formed by PANI, rGO, and AgNWs. This connected architecture improves energy dissipation and helps the material’s overall shielding effectiveness.

The obtained findings are consistent with the literature reports on hybrid EMI shielding systems that utilize PANI and carbon or metal nanofillers.

Most studies reported in the literature focus on polyaniline (PANI), graphene oxide (GO), or graphene-based systems for electromagnetic interference (EMI) shielding measurements. For example, incorporating GO into a PANI–polystyrene matrix significantly improved shielding efficiency, achieving 28 dB of reflection and 12 dB of absorption at only 1.5 wt% GO loading [12]. Similarly, polyaniline grafted onto γ-irradiated graphene oxide (GO-g-PANI) exhibited an EMI SE of 36.2 dB, which is significantly higher than that of pure GO, owing to a high grafting ratio and enhanced charge delocalization at the interface [13]. Graphene-based polyaniline (PANI) hybrids prepared by in situ polymerization exhibited a maximum reflection loss of −32.1 dB at 5.45 GHz and an effective absorption bandwidth of 5.62 GHz, confirming strong π–π interactions and efficient electromagnetic wave attenuation between graphene sheets and PANI chains [22]. Free-standing para-toluenesulphonic-acid-doped PANI/graphene-nanoplatelet films achieved over 95% shielding efficiency in the X-band region due to improved conductivity and interfacial polarization stemming from π–π conjugation between PANI chains and graphene sheets [75].

In contrast, PANI composites containing rGO or AgNWs for EMI shielding have been less frequently studied. Among previous reports, the system with S-doped RGO embedded in PANI (PANI/S-rGO/paraffin) showed the most balanced combination of absorption and reflection mechanisms, with a total shielding effectiveness of −22.5 dB, indicating that over 99% of incident EM waves were attenuated. This improvement results from the synergistic interaction between PANI chains and doped graphene sheets, which increases the dielectric constant (ε′), enhances dipole alignment, and introduces ohmic losses that convert into heat [8]. When aniline-functionalized rGO was added to polymerized matrices, the resulting composite achieved excellent microwave absorption of −47.1 dB at 9.6 GHz, emphasizing the importance of impedance matching and interfacial dipole polarization in EM attenuation [15]. Likewise, gold-coated PANI/rGO/paraffin systems exhibited 54% higher conductivity than pure PANI/paraffin at 12 GHz, along with increased dielectric loss, showing efficient conversion of EM energy into heat via interfacial polarization mechanisms [16]. The role of metallic nanofillers was confirmed by Fang et al., where AgNWs/PANI composite films reached SEA = 26.9 dB and SER = 10.8 dB at 11 vol% AgNWs, indicating that 99.8% of incident EM power was absorbed within the film due to increased electrical conductivity and multiple reflections inside the layered structure [14].

Ponnamma et al. reported that PCL-based nanocomposites containing PANI and rGO achieved EMI shielding efficiencies of 32–42 dB in the 8–13 GHz range [78]. Unlike our in situ-synthesized nanocomposites, their materials were produced by physically blending PANI and thermally reduced rGO within the PCL matrix.

In all existing literature, studies on EMI shielding of ternary PANI/rGO/AgNW composites are limited. Similarly to our findings, Suryaprabha et al. demonstrated that GO–PANI–Ag-coated cotton fabrics achieved an EMI shielding effectiveness of about 52 dB in the X-band range (8.2–12.4 GHz), which increased to 84.9 dB with additional coating layers [79]. The authors attributed this high performance to a combined effect of absorption, including internal material losses and multiple reflections within the material, where the absorption contribution (SEA) surpassed the reflection contribution (SER) due to strong interfacial polarization between GO and PANI and dielectric loss from Ag nanoparticles. Additionally, comparable results were reported by Das et al. The shielding efficiency increased from 8 dB after two coating cycles to approximately 34 dB after 18 cycles, as the dense deposition of RGO-Ga nanoparticles and PEDOT:PSS formed a continuous conductive network within the textile matrix [80].

However, flexible films with EMI shielding properties made by incorporating PANI/rGO–AgNWs hybrids into a PCL matrix have not yet been documented in the literature. The novelty of this approach lies in combining conductive polymer–graphene–silver networks within a polymer host, producing films that provide both mechanical flexibility and effective electromagnetic shielding.

## 3. Materials and Methods

### 3.1. Materials

Aniline (Py, p.a. 98%, Sigma-Aldrich, St. Louis, MO, USA), ammonium peroxydisulfate (APS, T.T.T. d.o.o., Sveta Nedelja, Croatia), polycaprolactone (PCL, Thermo Scientific, NJ, USA), hydrochloric acid (HCl, 32%, Fluka Chemie GmbH, Buchs, Switzerland), ethanol (C_2_H_5_OH, ≥99.8%, Fisher Scientific, Loughborough, UK), chloroform (CHCl_3_, Macron Fine Chemicals™, Gliwice, Poland), and deionized water were used as received without further purification.

### 3.2. Synthesis of PANI/rGO, PANI/AgNWs, and PANI/rGO-AgNWs Composites and Preparation of Their Films in PCL

Before fabricating the composites with PANI and PCL, rGO, silver nanowires (AgNWs), and their hybrid (rGO-AgNWs, 2:8) were prepared according to the method reported by Milenković et al. [81]. They were further used to prepare composites with PANI and PCL.

Flexible PANI/PCL-based films with nanofillers rGO, AgNWs, or rGO–AgNWs were produced using a two-step synthesis process. During the first step, polyaniline (PANI) was synthesized via chemical oxidative polymerization of aniline in an acidic medium, using ammonium persulfate (APS) as the oxidizing agent, in the presence of nanofiller. Aqueous solutions of APS (20 mmol in 20 mL of 1.2 M HCl) and aniline monomer (16 mmol in 20 mL of 1.2 M HCl) were simultaneously added to GO, AgNWs, or GO-AgNWs hybrid pre-dispersed in water (100 mL, 1 mg/mL). The mixture was stirred for 1 h at room temperature. The resulting precipitate was collected by filtration, washed with ethanol containing 1.2 M HCl, and dried under vacuum at 60 °C for 3 h to obtained nanocomposite powder samples entitled PANI/rGO, PANI/AgNWs, and PANI/rGO–AgNWs. Pristine PANI was prepared using the same procedure but without adding nanofillers.

In the second step, the nanocomposites (PANI/rGO, PANI/AgNWs, or PANI/rGO–AgNWs) were dispersed in a viscous PCL solution in chloroform to create flexible composite films with enhanced mechanical strength, denoted PANI/rGO/PCL, PANI/AgNWS/PCL and PANI/rGO–AgNWs/PCL. Specifically, 0.1 g of each nanocomposite was dispersed in 2.5 mL of PCL solution (prepared by dissolving 9.6 g of PCL in 75 mL of chloroform). The dispersions were homogenized until uniform, then cast into molds. The films were left to dry at room temperature until all the solvent had evaporated. A composite film of pristine PANI in PCL was prepared using the same procedure and denoted PANI/PCL.

### 3.3. Methods

Scanning electron microscopy (SEM) was used to examine the surface morphology of the PANI, PANI/GO, PANI/AgNWs, and PANI/GO–AgNWs samples. The analyses were conducted with a SCIOS 2 Dual Beam scanning electron microscope (Thermo Fisher Scientific, Waltham, MA, USA) equipped with an energy dispersive X-ray spectrometer (EDS). Measurements were taken at an accelerating voltage of 10 kV. EDS elemental mapping was also performed at 10 kV and a magnification of 5000×, with an acquisition time of 30 min. Before imaging, the samples were sputter-coated with a thin layer of gold (Au) to enhance surface conductivity and reduce charging effects.

Raman and Fourier Transform Infrared (FTIR) spectroscopies were applied to examine the molecular structure of the powder nanocomposites (PANI, PANI/GO, PANI/AgNWs, and PANI/GO–AgNWs) and to assess possible interactions among their components. Raman spectra were obtained from as-synthesized powder samples using a DXR Raman microscope (Thermo Fisher Scientific, Waltham, MA, USA) equipped with a 532 nm excitation laser. For each sample, three randomly selected spots were analyzed using a laser power of 2 mW, with an acquisition time of 10 × 10 s. FTIR spectra were recorded with a Nicolet iS20 spectrometer (Thermo Scientific, Waltham, MA, USA) equipped with a diamond ATR crystal. Approximately 5 mg of each nanocomposite powder was mixed with 100 mg of KBr, homogenized, and pressed into pellets before measurement. The spectra were collected at a resolution of 4 cm^−1^, averaging 16 scans per spectrum.

Thermal stability of the pristine and composite samples, along with their corresponding PCL-based films, was assessed using thermogravimetric analysis (TGA) on a TGA/DSC 3+ instrument (Mettler Toledo GmbH, Greifensee, Switzerland). About 3 mg of each sample was heated from 25 °C to 800 °C under a flow of 20 mL min^−1^ of oxygen, at a steady heating rate of 10 °C min^−1^. Each test was conducted twice to verify reproducibility.

Thermomechanical analysis (TMA) was conducted to evaluate the thermal and mechanical properties of the composite films using a TMA7 SDTA 2+ system (Mettler Toledo GmbH, Greifensee, Switzerland). Specimens measuring 4 × 4 × 1 mm^3^ were subjected to a temperature increase from 22 °C to 140 °C at 2 °C min^−1^ under dynamic loading conditions. The sinusoidal force oscillated between 0.05 N and 0.5 N with a cycle period of 6 s, in a nitrogen atmosphere flowing at 30 mL min^−1^.

To evaluate the surface wettability of the prepared films, contact angle (CA) measurements were performed using a Theta Lite goniometer (Biolin Scientific, Göteborg, Sweden). The sessile drop method was used, where a 6 μL droplet of deionized water (18.2 mΩ·cm) was gently placed onto each film sample surface (PCL, PANI/PCL, PANI/rGO/PCL, PANI/AgNWs/PCL, and PANI/rGO–AgNWs/PCL) with a microsyringe. Measurements took place under ambient lab conditions (25 °C). Water droplet images were recorded 5 s after deposition to allow the drop profile to stabilize. For each sample, the CA was measured at ten different points on the surface, with representative images shown. Data acquisition and analysis were carried out using the OneAttension software package (version 4.0.3).

EMI shielding effectiveness of composite PCL films is measured using samples measuring 20 mm × 30 mm, designed to cover the inner window of WR–90 waveguide adapters (15 mm × 25 mm). The S-parameters (S_11_ and S_21_) were measured with a vector network analyzer (VNA), specifically a Rohde & Schwarz ZVA 24 (Munich, Germany). The frequency range was 8–12 GHz. RF coaxial cables connected the WR-90 waveguide adapters to ports 1 and 2 of the Rohde & Schwarz ZVA 24 VNA. The composites were placed between two waveguide adapters, and the S_21_ scattering parameters were recorded as previously described [82].

For calculating EMI shielding effectiveness (SE), we used Equation (1):

SE (dB) = −20*log_10_(∣S_21_∣) (1)

where the SE of samples is calculated from the transmission coefficient (S_21_) measured with a VNA.

The sheet resistance of the samples was measured by the four-point probe method (JANDEL RM 3000).

Generative artificial intelligence (GenAI—ChatGPT) tools were used to generate sections of Scheme 1 and assist with grammatical editing of the manuscript.

## 4. Conclusions

The biodegradable PCL matrix combined with conductive PANI, rGO, and AgNWs offers a promising platform for developing lightweight, flexible, and environmentally friendly shielding films suitable for next-generation electronic and wearable devices. Findings in this study highlight that customizing the morphology and interfacial structure of hybrid 1D/2D filler systems provides an effective way to enhance the multifunctional properties of polymer-based EMI shielding materials. The results indicate that adding conductive nanofillers, either alone or in combination, changes the physicochemical properties and EMI shielding performance of the host polymer.

Comprehensive structural characterization (SEM, Raman, FTIR) confirmed uniform coating of rGO sheets and AgNWs with PANI, resulting in the formation of hierarchical 3D conductive networks. Thermal and thermomechanical analyses (TGA, TMA/DMA) showed improved thermal stability and stiffness across all composite systems compared to neat PANI/PCL, with the most notable enhancement observed in the quaternary PANI/rGO–AgNWs/PCL system, driven by synergistic interfacial interactions and restricted chain mobility.

The EMI shielding measurements in the X-band (8–12 GHz) showed that the PANI/rGO composite had a total shielding effectiveness (~7.2 dB). Adding AgNWs improved thermal and mechanical stability and influenced the uniform conductive network formed by PANI and rGO, causing a slight decrease in EMI shielding performance. The hybrid rGO–AgNWs structure provided a balanced but complex contribution, illustrating the delicate interaction between one-dimensional (AgNWs) and two-dimensional (rGO) conductive fillers within the polymer matrix.

## Figures and Tables

**Table 1:** Summary of TGA parameters for pristine and composite PCL-based films.

Sample	Tonset (°C)	Tmax (°C)	Residue at 800 °C (%)	Δm Steps (%)
PCL	≈315/395/438	437.9	0.01	≈82/8/10
PANI/PCL	≈66/352/488	487.9	0.1	≈4/71/25
PANI/rGO/PCL	≈67/354/481	480.6	0.7	≈5/69/26
PANI/AgNWs/PCL	≈68/364/499	499.4	3.4	≈3/81/13
PANI/rGO–AgNWs/PCL	≈66/308/386/402/495_(shoulder)_	495.0	2.5	≈4/9/40/44

## Data Availability

The datasets analyzed in the current study are available in the Zenodo repository (https://doi.org/10.5281/zenodo.17837867).

## Supplementary Materials

The following supporting information can be downloaded at: https://www.mdpi.com/article/10.3390/molecules30244693/s1. Figure S1. Thermogravimetric analysis of neat PCL film.
